# A125 DIAGNOSTIC ACCURACY OF ARTIFICIAL INTELLIGENCE IN THE DIAGNOSIS OF INTESTINAL METAPLASIA AND DYSPLASIA IN PATIENTS WITH BARRETT'S ESOPHAGUS: A DIAGNOSTIC TEST ACCURACY META-ANALYSIS

**DOI:** 10.1093/jcag/gwad061.125

**Published:** 2024-02-14

**Authors:** K Dadgar, L Mais, S Sangar, M Yaghoobi

**Affiliations:** Gastroenterology, McMaster University, Hamilton, ON, Canada; Gastroenterology, McMaster University, Hamilton, ON, Canada; McMaster University Faculty of Health Sciences, Hamilton, ON, Canada; Gastroenterology, McMaster University, Hamilton, ON, Canada

## Abstract

**Background:**

Diagnosing dysplasia in patients with Barrett’s Esophagus is crucial in preventing esophageal cancer but challenging in clinical practice. Artificial Intelligence (AI) could potentially be utilized during endoscopy to provide better diagnostic accuracy.

**Aims:**

The primary aim of this systematic review is to determine the diagnostic accuracy of AI in detecting intestinal metaplasia and dysplasia in adults with Barrett's esophagus using gastroscopy images.

**Methods:**

A comprehensive electronic search was conducted of cross-sectional studies examining the accuracy of AI in diagnosing intestinal metaplasia or dysplasia using endoscopic images. Study selection, data extraction and quality assessment were completed by two authors independently. When a study used several models, the model with the highest sensitivity was used in meta-analysis. The Quality Assessment of Diagnostic Accuracy (QUADAS-2) tool was used to assess risk of bias and applicability. Meta-analysis was performed using a bivariate model to obtain summary estimates of sensitivity, specificity, and diagnostic odds ratio.

**Results:**

Of the 1479 articles reviewed, 23 were included. The diagnosis of dysplasia by AI per endoscopic image obtained had a sensitivity of 0.92 (CI 0.85-0.96), specificity of 0.84 (CI 0.78-0.88) and diagnostic odds ratio (DOR) of 59 (CI 22-161). The diagnosis of dysplasia in volumetric laser endomicroscopy images interpreted by AI had an overall sensitivity of 0.83 (CI 0.70-0.91), specificity of 0.77 (0.67-0.85) and a DOR of 16 (CI 2-30). Subgroup analysis did not show a statistically significant difference when comparing studies conducted in Europe to those outside of Europe or studies published before 2020 to those published after 2020. A sensitivity analysis by removing the largest studies did not change the overall accuracy of AI.

**Conclusions:**

AI algorithms seem to be accurate at detecting the presence of intestinal metaplasia and dysplasia. Further research into using artificial intelligence should be carried out to evaluate its use in combination with endoscopist’s opinion as a clinical decision-making tool to target areas of dysplasia for biopsies.

**Funding Agencies:**

None

